# Food-Body-Mind study protocol: A mindfulness-based lifestyle intervention to promote whole child health among preschoolers from economically marginalized families

**DOI:** 10.1016/j.cct.2025.108146

**Published:** 2025-11-19

**Authors:** Jiying Ling, Tsui-Sui Annie Kao, Lorraine B. Robbins, Charis L. Wahman, Jean M. Kerver, Kenneth Resnicow, Nanhua Zhang, Hannah Lalonde, Yingcen Xie, Jennifer Baumgartner

**Affiliations:** a Michigan State University College of Nursing, USA; b Michigan State University Department of Counseling, Educational Psychology and Special Education, USA; c Michigan State University Department of Epidemiology and Biostatistics, USA; d University of Minnesota Division of Epidemiology & Community Health, USA; e Cincinnati Children’s Hospital Medical Center Department of Pediatrics, Division of Biostatistics & Epidemiology, University of Cincinnati College of Medicine, USA; f Program Director in the Clinical Research Branch, Division of Extramural Research, Basic and Mechanistic Research in Complementary and Integrative Health Branch, USA

**Keywords:** Mindfulness, Behavioral health, Emotional well-being, Mental health, Lifestyle, Preschool

## Abstract

Socioeconomic, racial/ethnic, and urban/rural disparities in mental, emotional, behavioral, and physical health are evident among U.S. preschoolers. To reduce these disparities, we plan to test our Food-Body-Mind intervention with preschoolers from racially/ethnically diverse, low-income, urban, and rural areas. The aims of the study are to determine the effects of the intervention on improving preschoolers’ behavioral (primary outcome is problem behaviors), mental, emotional, and anthropometric health outcomes, as well as their caregivers’ mental and cardiometabolic health from baseline to post-intervention to 12-month (Month 16) follow-up. Additionally, we will explore potential mediators (e.g., caregiver mindfulness, physical activity [PA], fruit/vegetable [F/V] intake, caregiver-preschooler relationship, caregiver coping, home environment, and household food insecurity) of the intervention effects. This cluster randomized controlled trial will include 50 daycare centers assigned to intervention (25 centers) or control (25 centers) conditions using a covariate-constrained randomization approach. A total of 400 (on average 8 per center) caregiver-preschooler dyads will be enrolled from 2024 to 2027. The 16-week mindfulness-based intervention includes three components: 1) school-based mindful eating and movement learning for preschoolers; 2) a home-based caregiver skill training on practicing mindful eating, movement, and parenting behaviors at home; and 3) a bridge between school and home in mindfulness learning and practice. Linear mixed-effect models will be used to determine the intervention effects, and structural equation modeling will be performed to examine potential mediators.

**Trial registration number:**

NCT06597474.

## 1. Introduction

Mental, emotional, and behavioral (MEB) disorders begin in early childhood, with one in six U.S. children under age 6 experiencing a mental health problem (e.g., anxiety, conduct disorder, attention-deficit/hyperactivity disorder [ADHD]) [1]. Yet only 8 % of these young children have received behavioral interventions or psychotherapy from a mental health professional [2]. Consequently, many MEB concerns during preschool age persist into adolescence [3], with one in six youth aged 10–19 years experiencing a mental health condition [4] and 19 % of U.S. high school students considering attempting suicide [5]. Moreover, MEB concerns in early childhood are linked to cardiometabolic profiles in later childhood [6]. Preschoolers in households with low socioeconomic status (SES) are nearly two times more likely to develop MEB and cardiometabolic problems than those from higher-income households [7–9]. Additionally, low SES is more strongly correlated with mental health problems in young children than in adolescents [10]. Optimal early childhood development confers lifelong benefits for education, productivity, economics, and population health [11,12]. Therefore, effective early-childhood interventions are critically needed to reduce existing MEB and cardiometabolic health disparities among preschoolers in low-SES households.

Because preschoolers spend much of their daytime in early care and education settings, these environments play a vital role in promoting MEB and cardiometabolic health [13]. Head Start, a federal program that promotes school readiness for millions of children, is an ideal setting for interventions targeting preschoolers from families with low-SES [14]. Although many school-based mental health interventions have been conducted with children, effects vary drastically, and few focus specifically on preschoolers and their caregivers [15,16]. Given that preschoolers are nurtured within home environments, meaningful caregiver involvement is crucial for fostering positive MEB and cardiometabolic health. Active family engagement has shown to almost triple the effects of school-based social-emotional interventions on improving preschoolers’ social skills (effect size Hedges’ *g* = 0.69 vs. 0.25) and reducing problem behaviors (*g* = −0.52 vs. 0.18; problem behaviors include internalizing [e.g., anxiety, fear, and sadness] [17] and externalizing [e.g., hyperactivity, aggressiveness, disruption] concerns [18]) [19]. However, previous home-based interventions (e.g., Family Check-Up [20], Triple P [21], and Incredible Years Group Parenting Programme [22]) primarily address school readiness and social-behavioral problems in at-risk preschoolers [23,24] and do not fully assess preschoolers’ mental and emotional well-being. Moreover, we found no program that comprehensively targets preschoolers’ health across both MEB and cardiometabolic domains.

Given the significant influence of school and home environments in shaping preschoolers’ optimal neurodevelopment [25], a comprehensive school- and home-based program is likely more effective for promoting MEB and cardiometabolic health than school-only or home-only approaches. To our knowledge, our proposed multi-component, school- and home-based Food-Body-Mind intervention is the first to target preschooler-caregiver dyads from low-SES urban and rural settings to improve dyadic mental and cardiometabolic well-being. In 2024, we conducted a single-group study (19 preschoolers, 18 caregivers, 3 teachers) to examine the feasibility, acceptability, and satisfaction of a 5-week Food-Body-Mind intervention [26]. The study yielded a 40 % enrollment rate, 0 % attrition, 95–100 % data collection rates, and 56–83 % intervention completion rates. Satisfaction rates were 78 % and 100 % among caregivers and teachers, respectively [26]. Building on the feasibility work, we will evaluate the effectiveness of the full 16-week Food-Body-Mind intervention in 50 Head Start centers.

## 2. Aims

The study has three specific aims: 1) Determine effects of the intervention on improving preschoolers’ behavioral (problem behaviors [primary outcome], social skills), mental (chronic stress by hair cortisol), emotional (sadness, fear, anger, positive affect), and anthropometric (body mass index [BMI] z-score, % body fat) health outcomes from baseline (0 month) to 4 months (immediate post-intervention) and to 16 months post-baseline (12-month follow-up) when compared to the control group; 2) Examine intervention effects on improving caregivers’ cardiometabolic (BMI, % body fat, blood pressure) and mental (stress, anxiety, depression) health from 0 to 4 months and to 16 months compared to the control; and (3) Explore the potential mediators (caregiver mindfulness, PA, F/V intake, caregiver-preschooler relationship, caregiver coping, home environment, and household food insecurity) of intervention effects.

## 3. Methods

### 3.1. Design and setting

**Fig. 1.**
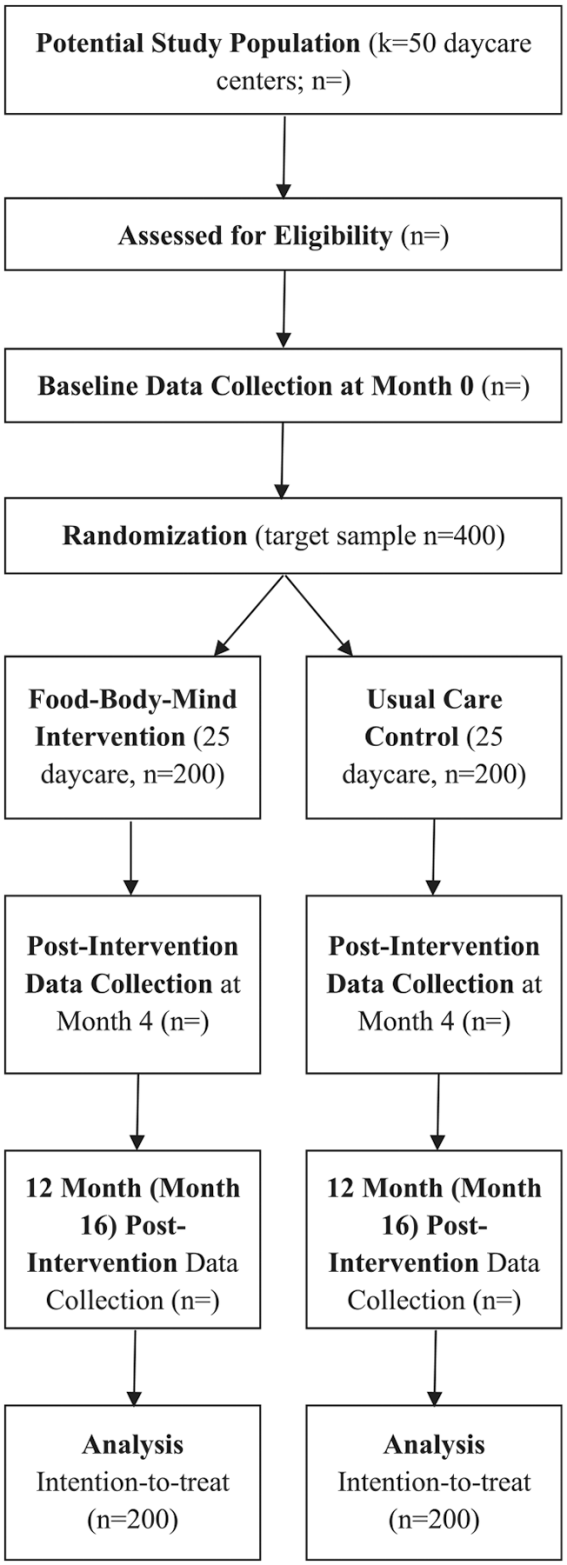
Flow diagram for the Food-Body-Mind study.

The cluster randomized controlled trial (ClinicalTrials.gov ID: NCT06597474) will be conducted in 50 Michigan Head Start daycare centers from 2024 to 2028, with participant enrollment from 2024 to 2027. Fifty Head Start daycare centers will be randomized into the intervention (*n* = 25: 8 urban and 17 rural daycare centers) or usual care control group (n = 25: 8 urban and 17 rural daycare centers). On average, eight caregiver-preschooler dyads will be recruited from each daycare center (total 400 dyads). All outcomes will be assessed at baseline (Month 0), post-intervention (Month 4), and 12-month follow-up (Month 16). Fig. 1 demonstrates the study flow diagram.

### 3.2. Recruitment and participants

Participant recruitment procedure includes: 1) we will identify daycare centers that are interested in participating and conduct a study meeting with daycare center director and supervisors to introduce the study and answer questions; 2) daycare center director and supervisors will then identify classrooms that are interested in participating; 3) we will provide participant recruitment training with communication scripts and recruitment flyers to family engagement specialists and classroom teachers to ensure consistency; 4) family engagement specialists and/or classroom teachers will hand out the recruitment flyer (including study activities on data collection and intervention participation, data collection incentives, contact information, and access to an enrollment and screening survey) and explain the study to each caregiver in person or virtually; and 5) interested caregivers will be asked to contact the study team for any questions and complete the enrollment and screening survey by either clicking the survey link or scanning the QR code. Trained participant recruiters will attend parent orientation meetings and family fun nights in person or remotely to introduce the study to caregivers. Recruitment flyers will be posted around Head Start centers and/or on Head Start social media sites. Trained participant recruiters will also conduct in-person recruitment during daycare drop-off and pick-up times.

Participants must meet all inclusion criteria to participate.

#### Inclusion criteria.

Preschoolers must: 1) have parental consent, 2) have child verbal assent if being 5 years, 3) be 3–5 years, and 4) be enrolled in a Head Start program. Caregivers must: 1) provide consent, 2) be the primary adult caregiver (≥18 years) for the preschooler. Primary caregiver refers to the one person most responsible for providing care to the preschooler on a daily basis; 3) have at least weekly Internet access using a smartphone, tablet, or a computer; and 4) be willing to use Facebook or the “Food-Body-Mind” private website for participating in the intervention. Daycare teachers are eligible to participate when they are the adult teachers (≥18 years) and willing to participate in data collection by providing informed consent.

#### Exclusion criteria.

Exclusion criteria for preschoolers are: 1) children with a motor disability or impairment (e.g., cerebral palsy, spinal cord injury, lost or damaged limb) that prevents from participating in any PA; 2) children with a diagnosed medical condition (e.g., phenylketonuria, pediatric malabsorption syndrome) that requires a restricted diet and precludes dietary changes, particularly increased F/V consumption; or 3) children with a diagnosed neurodevelopmental disorder (e.g., autism spectrum disorder, level 3) that causes severe difficulties in communication and social interaction. There will be no exclusion criteria for caregivers or teachers, as our primary focus is preschoolers and caregivers/teachers serve in support roles.

### 3.3. Randomization

We will apply a covariate-constrained randomization schema to ensure that the intervention and control groups are balanced with respect to urban/rural setting, daycare sizes, and preschoolers’ ethnicity/race distributions at baseline. Immediately after baseline data collection, the study blinded statistician will generate 100,000 possible simple random allocation schemes. For each allocation scheme, balance will be estimated on the selected baseline characteristics according to the absolute difference. A constrained randomization space containing 10 % of the best allocations will be chosen, and one allocation scheme will be randomly selected from this constrained randomization space to determine the composition of the two groups.

### 3.4. Intervention

The Food-Body-Mind intervention is grounded in the Actor-Partner Interdependence Model (APIM) [27], the Allostatic Load Model [28], and the Transactional Theory of Stress and Coping [29]. The APIM has been used in lifestyle behavioral and mental health interventions to better understand intergenerational interactions and relationships inside a family [27]. The APIM demonstrates bidirectional relationship between preschoolers and caregivers: 1) actor effects within preschoolers or caregivers (how individuals influence their own outcomes), and 2) partner effects between preschoolers and caregivers (how family members influence one another) [27]. The intervention is grounded in this model to reinforce bidirectional partner effects: caregivers shape preschoolers’ behaviors through the home environment, and preschoolers, in turn, influencing caregivers’ parenting behaviors at home via our school-home connection component.

According to the Allostatic Load Model [28] and the Transactional Theory of Stress and Coping [29], stress process is an interaction between an individual and environment. In response to stress, individuals first cognitively appraise potential external stressors according to harm, threat, or challenge, and their means to cope with the stressor. Based on these initial appraisals, individuals respond with different coping strategies including problem-focused, emotion-focused, and avoidant coping. Problem-focused coping focuses on applying cognitive and behavioral efforts to change a stressful situation in a positive way [29]. Emotion-focused coping involves regulating difficult emotions such as anger, fear, anxiety, depression, frustration, and sadness that are associated with stress [29]. Avoidant coping can be a maladaptive form of coping demonstrated by denying or avoiding the stressful situations or feelings to temporarily reduce stress [30]. When the selected coping strategies fail to manage external stressors, the cumulative burden of chronic stress (allostatic load) can lead to poor health outcomes such as obesity, cardiovascular diseases, anxiety, and depression [31]. Healthy lifestyles including PA and healthy diet are associated with lower allostatic load, resulting in better cardiometabolic and mental health [32]. Building on these theories and hypotheses, the Food-Body-Mind intervention will focus on the connections among PA, F/V intake, and mental health to improve both preschoolers’ and caregivers’ adaptive coping strategies such as mindful eating and movement instead of maladaptive coping strategies such as emotional eating as a mechanism to enhance both their MEB and cardiometabolic health.

The 16-week Food-Body-Mind intervention (see Table 1) includes 3 components: 1) a school-based mindfulness component delivered by daycare teachers to equip preschoolers with knowledge and skills in mindful eating and movement; 2) a home-based mindfulness component delivered by trained research interventionists to increase caregivers’ skills in practicing mindful eating, movement, and parenting behaviors at home to foster a more positive, mindful, and healthy home environment; and 3) a school learning and home practice connection in mindfulness learning and practice. To reduce potential feelings of insecurity and anxiety, all daycare teachers will be trained to deliver the school-based program to all preschoolers in each intervention classroom, but data collection will occur only among the study preschoolers with parental consent. After the intervention, participating classrooms will retain the intervention supplies (e.g., an iPad with the child program, breathing balls, and food taste test supplies) and may continue the program with new students at their discretion. Control classrooms will receive teacher training and intervention supplies after the study ends.

We will support caregivers in preparing healthy meals at home by providing each caregiver with our well-tested “Tasty Healthy Cookbook,” which features budget-friendly recipes for healthy breakfasts, family meals, quick fixes, kid-friendly snacks, and occasional sweets. The cookbook was designed to meet the needs of families with low-SES, who may face tight budgets, limited time, and fewer opportunities to build cooking skills. It emphasizes ‘spend less, shop healthy’ strategies and offers many slow-cooker options that require minimal preparation and skills yet yield nutritious meals. Each preschooler-caregiver dyad will also receive two durable MyPlate portion plates (one adult, one child) to guide appropriate portions and food groups at home. To connect families with local supports, each intervention family will receive a community resource booklet listing free or low-cost healthy and mindful living resources (e.g., food pantries, farmers’ markets, community gardens, parks and trails, family activities) as well as accessible, vetted online resources. To support maintenance of effects, intervention caregivers will receive a brief motivational text message every Monday from the end of the intervention through the 12-month follow-up (Month 16).

### 3.5. Control

Preschoolers assigned to the control group will receive usual daycare activities during the intervention period. After the 12-month (Month 16) follow-up data collection, each control family will receive all intervention supplies including the “Tasty Healthy Cookbook,” “MyPlate” plates, and a breathing ball, as well as the program manual on how to use the intervention supplies and a community resource booklet. Moreover, a virtual caregiver meeting on mindful eating, movement, and parenting will be provided to all caregivers who are interested.

### 3.6. Data collection and measures

At each time point, data collection will include four components: 1) an online survey completed by caregivers and daycare teachers; 2) in-person data collection to measure dyads’ height, weight, % body fat, and skin carotenoid, as well as caregivers’ blood pressure; 3) ActiGraph (wGT3X-BT) accelerometer worn for 7 days to assess both preschoolers’ and caregivers’ PA (light PA, moderate to vigorous PA [MVPA]); and 4) hair sample collection to assess cortisol (as an indicator for chronic stress in the study) from a subgroup of preschoolers whose caregivers provided informed consent. Because this is a large-scale, community-based trial with young children and their caregivers from low-SES households, we intentionally selected noninvasive measures that provide reliable and valid assessments of mental and cardiometabolic health. We will not measure blood pressure in preschoolers due to inaccuracy from movement observed in our prior trial [33] and based on our pediatrician’s input indicating limited clinical significance of blood pressure screening in this age group in community settings. The study Data Manager and trained data collectors (blinded to group assignment) will be available by phone or in-person to answer questions. Table 2 depicts the measures used in this study and each measure’s reliability and validity. The schedule of assessment is demonstrated in Table 3.

**Preschoolers’ behavioral health** (problem behaviors and social skills) will be assessed using the Preschool and Kindergarten Behavior Scales-Second Edition [PKBS-2] [18]. This 76-item 4-point Likert scale contains two subscales: problem behaviors (42 items, *primary outcome*) and social skills (34 items). The four response choices for each item are never, rarely, sometimes, and often. Total raw scores will be calculated and converted to standard scores following the age-appropriate conversion tables [18]. The PKBS-2 will be completed by caregivers and teachers, with teacher-rated problem behaviors as the primary outcome.

**Preschoolers’ chronic stress** will be measured by hair cortisol. Hair sample collection is optional and caregivers may decline without impact on other study activities. With parental consent, hair samples will be collected by trained staff. A proximal 3-cm segment hair sample will be cut from 3 to 4 locations at the posterior vertex of each preschooler’s head with a stainless-steel styling shear. The total amount of hair to take is roughly half of the diameter of a pencil. Hair samples (labeled at the root ends furthest away from the head) will be stored in an aluminum foil pouch at room temperature. The hair samples will be analyzed by the Child Study Center lab at the Yale University using the enzyme immunoassay (Arbor Assays DetectX) approach to extract cortisl level [34].

**Preschoolers’ emotional well-being** (sadness, fear, anger, positive affect) will be assessed by the NIH Toolbox [35]. We will use the 7-item Sadness Parent Report Fixed Form to assess sadness, the 6-item Fear-Over Anxious Parent Report Fixed Form to assess fear, the 9-item Anger Parent Report Fixed Form [35] to assess anger, and the 9-item Positive Affect Parent Report Fixed Form to assess positive affect. The scales for assessing sadness, fear, and anger are 3-point Likert scales with response choices ranging from never or not true to often or very true. The positive affect scale is a 5-point Likert scale with responses from not at all to very much. A total raw score will be calculated and then transformed to the uncorrected T-scores (mean = 50, SD = 10).

**Caregivers’ mental health** (stress, anxiety, depression) will be assessed by the 10-item Perceived Stress Scale [36] and 4-item Patient Health Questionnaire ([PHQ]-4) [37]. The Perceived Stress Scales is s 5-point Likert scale with response choices of never to very often, and PHQ-4 is a 4-point Likert scale with responses ranging from not at all to nearly every day. A total raw score will be calculated for both scales.

#### 3.6.1. Dyads’ % body fat and BMI

Trained data collectors will measure dyads’ height (in cm) using the ShorrBoard^®^ Stadiometer, weight (in kg) and % body fat using the InBody 270 body composition analyzer following the National Health and Nutrition Examination Survey (NHANES) measurement protocol [38]. Height, weight, and % body fat will be measured without shoes, socks, or bulky clothes. When measuring % body fat, each participant’s biological sex, age, and height will be manually entered into the scale. After the setup, each participant will be instructed to step on the scale surface and align feet with the scale electrodes. Two measurements will be taken and averaged. If the two measurements differ by ≥0.5 cm for height, ≥0.5 kg for weight, and ≥ 1 % for % body fat, a third measurement will be taken and then the two closest measurements will be used. Preschoolers’ BMI for age and sex will be determined using the online SAS program for Centers for Disease Control and Prevention (CDC) Growth Charts [39]. Caregivers’ BMI will be calculated using (weight *kg*/height *m*^*2*^).

**Caregivers’ blood pressure** will be measured using the SunTech CT40 [40]. Blood pressure measurement will follow the the procedures in the Seventh Report of the Joint National Committee on Prevention, Detection, Evaluation, and Treatment of High Blood Pressure [41]. Three measurements of blood pressure will be taken at 30-s interval and averaged for recording.

**Dyads’ F/V intake** will be assessed using skin carotenoids (0–850) via Veggie Meter^®^ [42]. The Veggie Meter is a non-invasive portable spectroscopy-based device. Caregiver ring finger and preschooler index finger will be used to obtain an average of three measurements of skin carotenoids [43]. Two-question survey adapted from the National Cancer Institute’s Eating at America’s Table Study All-Day Screener [44] will also be used to assess dyads’ F/V intake. Each survey item has 10 response choices ranging from never to 5 or more times per day.

**Dyads’ PA** will be measured using the Actigraph (wGT3X-BT) [45] worn by both preschoolers and caregivers on hip. Before distributing the ActiGraph to dyads for wearing, we will use the ActiLife software to initialize each ActiGraph and set it to begin data collection after dyads receive the ActiGraph from data collectors. When distributing the ActiGraph to dyads, the “superhero with a magic belt” story will be shared with preschoolers to encourage them to wear, and written wear instructions will be provided to caregivers: right hip (attached to belt) from time getting out of bed in AM to going to sleep at night for seven consecutive days (not worn bathing/swimming). To improve compliance with wearing accelerometers, an auto text message reminder will be sent to caregivers every morning at 7 AM.

#### 3.6.2. Psychosocial mediators

Caregivers will complete an online survey via Qualtrics to assess caregiver-child relationship (Child-Parent Relationship Scale) [46], home eating and PA environment (Family Nutrition and Physical Activity Screening Tool) [47], household food insecurity (U.S. Household Food Security Survey Module) [48], caregivers’ mindfulness (Mindful Attention Awareness Scale) [49], and coping (Brief COPE) [50].

### 3.7. Process evaluation

Intervention attendance for all components will be documented. We will record: 1) caregiver task-completion status (4 tasks/week, total 56 tasks in 14 weeks; reach, dose delivered and received); 2) caregiver meeting attendance and whether meeting delivered according to protocol (3 group meetings; reach and dose delivered); 3) preschooler school program attendance (reach, 2 sessions/week, total 32 sessions in 16 weeks), engagement (preschooler is present for the whole session or not, dose received), and completion status of preschooler letters (dose received). Each caregiver will complete a short meeting evaluation survey (dose received) after each group meeting. Two multiple-choice questions related to the preschooler letters each week will be posted on the social media site to obtain caregivers’ responses to preschooler letters (dose received).

To evaluate intervention implementation fidelity, our independent Process Evaluators will conduct observations on various sessions delivered at multiple sites across study years. In each year, two independent Process Evaluators will observe two randomly sampled sessions per intervention daycare classroom (1 session at weeks 1–4 and the other session at weeks 8–12) to evaluate the adherence to intervention protocol of the school-based program. An additional process evaluation will be conducted when teacher turnover and transition occur in a certain classroom. Daycare staff turnover data will be recorded as ‘yes or no’ and timing when the turnover occurs. In addition, two independent Process Evaluators will observe one randomly selected meeting from the two meeting slots for each group meeting in each year to evaluate the meeting adherence to intervention protocol. During the intervention implementation, we will also record data on 1) participants who discontinue study intervention early with specific reasons, 2) potential reasons for early termination, and 3) adverse events occurred and reported.

### 3.8. Sample size determination

Based on literature [51] and our pilot studies [26,33,52–54], we assume an intraclass correlation coefficient (ICC) of 0.03 among daycare centers and an ICC of 0.02 among classrooms. Because some daycare center variation will be accounted for when classroom is also included as a random effect and vice versa, we expect the combined ICC to be 0.04, slightly smaller than the sum of 0.03 and 0.02. We expect to achieve a small-to-medium effect size of 0.35 for the selected primary outcome of preschoolers’ problem behaviors rated by teachers from baseline to immediately post-intervention, with a significance level of 0.05, sample size of 50 daycare centers (on average 8 dyads/center, range 5–14, 400 dyads) can achieve at least 86 % power.

### 3.9. Data analysis

All data analyses will be performed using IBM SPSS Statistics 27, SAS 9.4, or Mplus version 8.8. A *p*-value ≤0.05 will indicate statistical significance. All analyses will use intention-to-treat principle. The unit of randomization is ‘daycare center,’ so unit of analysis will be participants nested within each daycare center and each classroom. Comparisons of baseline demographic characteristics between groups will be made using two-sample *t*-tests for continuous variables and Chi-square tests (or Fisher’s exact tests) for discrete variables. If group differences are observed, relevant variables will be treated as covariates in post-intervention and follow-up analyses. To evaluate potential attrition bias, participants who drop out will be compared to those who remain in the study on demographics as well as baseline outcomes. Any characteristics or outcomes related to attrition will be included as covariates in analyses.

#### 3.9.1. Missing data

Statistical models will incorporate missing at random mechanisms [55] and derived inferences using full information maximum likelihood methods. For methods that require complete data, we will impute missing values using multiple imputations (*n* = 20) with PROC MI in SAS and IVEware SAS macro. Summary analyses from the 20 imputations will then be performed using SAS PROC MIANALYZE. To further examine the effects of missing data on results, we will perform sensitivity analysis to examine if the analysis results vary between non-imputed data and imputed data.

#### 3.9.2. Aim 1 & 2

Separate linear mixed-effect models will be used to determine the intervention effects on each outcome. Each model will include an interaction between group (intervention vs. control) and time (Month 4 and Month 16), baseline outcome, subject level random effects to account for repeated measures on the same subject over time, and cluster-specific random effects. Potential confounders that are identified in the baseline group comparisons and the cohort attrition analysis will be included in the model. We will also examine potential moderators (e.g., sex, ethnicity/race) of the intervention effects. In case there is a significant interaction, we will conduct post-hoc analyses to examine how the intervention effects differ at different levels of baseline outcome. Effect size Cohen’s *d* and 95 % confidence interval will be calculated.

#### 3.9.3. Aim 3

For the outcome constructs with multiple survey items, we will create composite scores (latent variables) based on results from confirmatory factor analysis. We will apply the 5-step structural equation modeling procedure: model specification, model identification, parameter estimation, model evaluation, and model modification. Hypothesized relationships (model specification) are driven by theoretical models. Model identification will be determined to make sure the model can be identified. A covariance matrix will be used for analysis, and parameter estimation will apply full information maximum likelihood approach. Both standardized and unstandardized coefficients will be calculated. Model fit will be evaluated via Tucker-Lewis Index (TLI ≥0.90), Comparative Fit Index (CFI ≥0.90), standardized root mean squared residual (SRMR ≤0.08), and root mean squared error of approximation (RMSEA ≤0.06) [56]. If model does not fit the data well, model modification will be conducted. Theoretical framework and literature results will be considered when modifying the model.

## 4. Discussion

Food-Body-Mind study is a cluster RCT aimed to determine the effects on improving mental and cardiometabolic well-being in preschoolers and their caregivers from low-SES backgrounds. This study design builds on multiple pilot clinical trials demonstrating the feasibility and preliminary efficacy of mindfulness-based lifestyle interventions [26,33,52–54]. An initial 10-week lifestyle program tested with 69 preschooler-caregiver dyads showed feasibility and preliminary efficacy (Cohen’s *d* = −0.30 for reducing BMI z-score; *d* = 0.40 for increasing F/V intake; and *d* = 0.42 for increasing MVPA among preschoolers) [54]. From 2020 to 2024, three additional pilots (total *n* = 402 dyads) examined integrating mindfulness into the lifestyle program and delivering the caregiver component using a private website [33,52,53]. Results supported feasibility and suggested preliminary benefits for cardiometabolic health (decreased BMI, % body fat, blood pressure), healthy lifestyle behaviors (increased F/V intake, skin carotenoids), food insecurity, and the home environment. However, effects on dyadic mental health and long-tern sustained effects remain unknown. Therefore, conducting this fully powered clinical trial is necessary to evaluate the intervention’s short- and long-term sustained effects on mental and cardiometabolic health. This study will also identify theoretical mediators that can help explain the interconnections among food, body, and mind.

During implementation, we may face some challenges in recruiting and retaining families from low-SES backgrounds, variability in implementation fidelity across Head Start centers, missing or incomplete measures, and caregiver burden and limited access to resources. To mitigate these possible risks, we will 1) use multi-pronged recruitment through teachers and family-engagement staff, flexible scheduling, modest incentives, and frequent check-ins; 2) provide standardized training, manuals, and fidelity checklists with ongoing observation and feedback; 3) employ child-friendly assessment protocols, repeat or home-based data collection when needed, and robust missing-data methods (e.g., multiple imputation); 4) minimize caregiver burden through brief, virtual delivery, make-up sessions, and a resource booklet linking families to free or low-cost community options. Collectively, these contingency plans are designed to preserve implementation fidelity, reduce bias from missing data, and support equitable participation across diverse urban and rural settings.

The study has a few potential limitations including participants cannot be blinded to group assignment, and results may not be generalizable to preschoolers not attending daycare centers or other states in the U.S. Moreover, because the sample will comprise families from low-SES backgrounds, results may not generalize to children or families with different SES. Although language is not an inclusion/exclusion criterion, the current English-language data collection and intervention materials may limit participation for families who do not speak English, despite Head Start’s available interpretation services. This trial is not designed to modify the school environment or directly address family access to foods and activities; rather, it tests the effectiveness of a teacher-implemented child program with active caregiver engagement. Despite these possible limitations, the trial employs a rigorous cluster randomized design that accounts for random cluster effects and reduces contamination, with a large, community-based sample drawn from both urban and rural settings. Construct validity is strengthened through noninvasive biomarkers (hair cortisol, skin carotenoids, blood pressure) and objective measures (ActiGraph accelerometry, ShorrBoard^®^ stadiometry, bioelectrical impedance analysis). The intervention is theory-driven and integrates coordinated components across school, home, and the school-home connection, addressing multiple, mutually reinforcing contexts.

This trial will generate evidence on both immediate and sustained effects of a mindfulness-based lifestyle intervention on MEB and cardiometabolic health among preschoolers and their caregivers. By simultaneously engaging the school, home, and school-home connection, the study tests a multi-context approach that reflects real world conditions and may be more durable than single-setting programs. The findings can inform clinical and public health guidance for early prevention. If effective, Food-Body-Mind offers a feasible and developmentally appropriate model that Head Start and other early care and education systems could adopt to address MEB concerns alongside diet, PA, and metabolic domains that are rarely targeted together in early childhood. If effects are maintained at follow-up, the model would support policy arguments for investment in integrated, mindfulness-based lifestyle programs within early childhood systems.

Additionally, the study will contribute mechanistic insights. By examining theoretical mediators (e.g., caregiver mindfulness, coping, caregiver-child relationship, home environment) and objective/noninvasive markers (e.g., hair cortisol, skin carotenoids, blood pressure, accelerometry), the trial can clarify the food-body-mind linkage and identify leverage points for promoting well-being in early childhood. Following the trial, priorities include evaluating cost-effectiveness and pursuing longer-term follow-up into the elementary school years.

## Figures and Tables

**Table 1:** 16-week Food-Body-Mind intervention (three components: school-based, home-based, school-home connection).

Component	Dose	Theme, objectives, and description
*School-based* min*dfulness component for preschoolers*	20 min*/wk., 16 weeks*	*Mindful Eating – “Eat My ABCs ”* ***Objective:*** *Increase mindful eating knowledge and skills, expose children to healthy food options Each lesson structure:* ***Beginning:*** *Children are learning one fruit and one vegetable using 4 senses: see, feel, hear, smell*. ***Middle:*** *Children are practicing* min*dful eating to taste each fruit and vegetable*. ***End:*** *Reinforce the connection among food, body, and* mind: *benefits of healthy foods (*e.g.*, F/V) to our body (*e.g.*, help grow, become strong), mind (*e.g.*, be happy), and health (*e.g.*, not get sick)*.
	*20 min/wk., 16 weeks*	*Mindful Movement - “Walk My ABCs”* ***Objective*** *: Improve relaxation technique knowledge, skill, and physical activity Each lesson structure:* ***Beginning:*** *Warm up with jumping jacks and stretching and make body-*min*d connection (*e.g.*, connect movement to body and feelings)*. ***Middle:*** *Practice yoga poses (*e.g.*, airplane, butterfly, candle, down dog,* etc.*) and learn mindful breathing exercises with a breathing ball*. ***End:*** *Make the body-mind connection and reflect on the benefits of participating in physical activity and* mindful *movement* (e. g.*, help grow, be strong, stay healthy, sleep better, feel happy)*.
*Home-based mindfulness component for caregivers*	*1 flyer, 1 video, and 4 tasks/wk., 14 weeks*	*Social media-based program* ***Objective****: Foster a supportive virtual community among participating caregivers and provide parenting, health, and mindfulness information. One weekly electronically retrievable flyer and one short video modeling the daily* min*dful parenting practices at home by peer caregivers are shared each week*. *Weekly habit-formation tasks:* ***Create a healthy family****: post about a healthy meal made for their family, or a physical activity they helped child engage, or ways of practicing mindful eating and movement at home*. ***Raise a happy family****: post about a life management tip they used to manage life and stress, or a strategy used to improve parent-child relationship*. ***Leave a positive comment****: positively respond to one other person’s post to build a virtual supportive community*. ***Take a quiz:*** *take a short online quiz to reinforce information and strategies learned*.
	*3 texts/wk., 16 weeks* *1 text/wk., 12 months after intervention*	*Weekly motivational text messaging* ***Objective:*** *increase motivation to make healthy and mindful changes at home*. *A text message is sent on Monday, Wednesday, and Friday: 1) Monday messages will focus on parenting and family, 2) Wednesday messages on lifestyle changes and habit formation, and 3) Friday message on stress management and mindful practice. Weekly motivational text messages will continue during follow-up, once a week, on Monday*.
	*60 min/meeting, 3 meetings*	*Virtual group meetings at week 1, 8, 16* ***Objective:*** *strengthen child-parent relationship, connect caregivers for peer support, and enhance mindfulness practices at home. Each meeting will contain a 20-mintues yoga practice and discussion on mindful eating, mindful movement, and mindful parenting*.
*School-home connection*	*2 letters/wk., 16 weeks*	*Preschooler letters* ***Objective:*** *connect preschooler learning in daycare with caregiver practice at home, improve caregiver-preschooler relationship* *Each week at daycare, preschoolers will create two letters using stickers regarding the food and activities they tried, liked, and want to try at home*. *Caregivers are encouraged to discuss the letters with their preschooler and offer the food and activities at home. Caregivers will also be asked to answer two questions about the letters each week: (1) what foods listed in your child’s letter did you provide? (2) What activities listed in your child’s letter did your family try?*

**Table 2:** Study outcomes and measures.

Variable	Measure and Description	# Items	Time	Reliability/Validity
**Aim 1 (Preschooler)**				
Behavioral health	Preschool and Kindergarten Behavior Scales-Second Edition (PKBS-2) [18] has two subscales: social skills and problem behaviors (**Primary outcome**).	76	12 min	**Internal consistency reliability** *a* = 0.75–0.97 [57] **Test-retest reliability** *r* = 0.58–0.87 **Validity** predicted autism spectrum disorders
Chronic stress	Hair cortisol concentration. Hair samples will be analyzed at Yale Child Study Center [34].	N/A	5 min	**Validity** *r* = 0.29–0.32 related to salivary cortisol [58]
Sadness	Sadness Parent Report Fixed Form [35]	7	2 min	**Internal consistency reliability** *a* = 0.77 **Convergent validity** = 0.38 [35]
Fear	Fear-Over Anxious Parent Report Fixed Form [35]	6	2 min	**Internal consistency reliability** *a* = 0.79 **Convergent validity** = 0.60 [35]
Anger	Anger Parent Report Fixed Form [35]	9	2 min	**Internal consistency reliability** *a* = 0.85 **Convergent validity** = 0.64 [35]
Positive affect	Positive Affect Parent Report Fixed Form [35]	9	2 min	**Internal consistency reliability** *a* = 0.92 **Convergent validity** = 0.95 [35]
BMI z-score	Calculated from height and weight using CDC growth charts [39] **Height:** Infant/Child/Adult ShorrBoard^®^ Measuring Board Stadiometer **Weight:** InBody 270 body composition analyzer	N/A	3 min	**Specificity** = 0.93 **Sensitivity** = 0.73 [59]
% Body fat	InBody 270 body composition analyzer applies bioelectric impedance technology to assess body composition including % body fat.	N/A	2 min	**Validity** highly correlated with the dual-energy X-ray absorptiometry method among children (*r* = 0.97) [38]
**Aim 2 (Caregiver)**				
BMI	Calculated using (weight *kg*/height *m*^*2*^) [60] Measures are the same as those used in preschoolers	N/A	3 min	**Specificity** = 0.97 **Sensitivity** = 0.42 [61]
% Body fat	InBody 270 body composition analyzer	N/A	2 min	**Validity** highly correlated with the dual-energy X-ray absorptiometry method among adults [62]
Blood pressure	SunTech CT40 Blood Pressure device	N/A	5 min	**Validity** achieved A/A grade of the British Hypertension Society protocol and reached the standard of the Association for the Advancement of Medical Instrumentation (AAMI)/the International Organization for Standardization (ISO) [40]
Stress	Perceived Stress Scale (PSS) [36]	10	2 min	**Internal consistency reliability** *α* = 0.74–0.91 **Test-retest reliability** *r* = 0.72–0.88 **Validity** *r* = 0.66–0.72 correlated with Hospital Anxiety and Depression Scale, *r* = 0.73 related to State-Trait Anxiety Inventory, *r* = 0.69 correlated with Post Traumatic Stress-Arousal Scale [63]
Anxiety & Depression	Patient Health Questionnaire ([PHQ]-4) [37]	4	1 min	**Validity** *r* = 0.80 with mental health **Construction validity** 2-factor structure [37]
**Aim 3 (Preschooler-Caregiver Dyads)**				
Physical activity (min/h)	7-day ActiGraph wGT3X-BT accelerometer **Cut-points for preschoolers:** sedentary activity (≤ 37 counts/15 s), light (38–419), moderate (420–841), & vigorous (≥842) [45] **Cut-points for caregivers:** light (0–2689 counts/60 s), moderate (2690–6166), & vigorous (≥ 6167) [64]	7-day	5 min	**Preschoolers:** **Reliability** = 0.69–0.84 [65] **Validity** *r* = 0.66 with observational system [66] **Caregivers:** **Reliability** ICC = 0.97–0.99 [67] **Validity** *r* = 0.81 with oxygen consumption [68]
F/V intake	Skin carotenoid level via Veggie Meter [42,69,70] 2-question survey adapted from the National Institute of Health Eating at America’s Table Study All-Day Screener	N/A	2 min	**Intra-individual variation of triple measurements** 6.8 % [43] **Validity** *r* = 0.81 related to serum carotenoid level [71], *r* = 0.48 related to self-reported F/V intake [43] Used in interventions to evaluate fruit/vegetable changes [44]
Caregiver-preschooler relationship	Child-Parent Relationship Scale (CPRS) [46,72] is a 5-point Likert scale with 2 subscales: conflict and closeness.	15	3 min	**Internal consistency reliability** *α* = 0.64–0.84 [46] **Construct Validity** 2-factor structure [73]
Caregiver mindfulness	Mindful Attention Awareness Scale [49] is a 6-point Likert scale to assess trait mindfulness.	15	3 min	**Reliability** 0.92 [74] **Construct validity** 1-factor structure [74]
Caregiver coping	Brief COPE [50] has 14 factors that can be grouped to 3 subscales: problem-focused coping, emotion-focused coping, and avoidant coping.	28	5 min	**Internal consistency reliability** *α* = 0.25–1.00 [75] **Test-retest reliability** = 0.32–1.00 [75] **Construct validity** 14-factor structure [50]
Home environment	Family Nutrition and Physical Activity Screening Tool [47]	20	4 min	**Internal consistency reliability** a = 0.75 **Validity** correlated with child BMI [47]
Household food insecurity	U.S. Household Food Security Survey Module [48]	19	4 min	**Sensitivity** = 32 % **Specificity** = 90 % [48]
Demographics	Socio-demographic questionnaire (e.g., age, sex, race, family income, education, marital, employment)	12	2 min	N/A

**Table 3:** Schedule of assessment.

Assessments	T0 Recruitment	T1 Baseline (Month 0)	T2 Postintervention (Month 4)	T3 12-month follow-up (Month 16)
Parent Informed Consent	X			
Child Verbal Assent	X			
Teacher Consent		X		
Screening Survey	X			
Family Contact Information Survey	X		X	
Dyad Demographic Information	X			X
Teacher Demographic Information		X		
**Aim 1 (Preschooler)**				
*Child Behavior Scales (completed by caregiver and teacher) – PKBS-2		X	X	X
Child Chronic Stress – Hair Cortisol		X	X	X
Child Sadness Survey		X	X	X
Child Anxious Survey		X	X	X
Child Anger Survey		X	X	X
Child Positive Affect Survey		X	X	X
Child BMI z-score		X	X	X
Child % Body Fats		X	X	X
**Aim 2 (Caregiver)**				
Caregiver BMI		X	X	X
Caregiver % Body Fat		X	X	X
Caregiver Blood Pressure		X	X	X
Caregiver Stress Survey		X	X	X
Caregiver Anxious & Depression Questionnaire - PHQ-4		X	X	X
**Aim 3 (Preschooler-Caregiver Dyads)**				
Dyad Physical Activity – ActiGraph wGT3X-BT		X	X	X
Dyad F/V Intake Survey		X	X	X
Dyad Skin Carotenoids		X	X	X
Caregiver Mindfulness Survey – MAAS		X	X	X
Child-Parent Relationship Scale – CPRS		X	X	X
Caregiver Coping Survey – Brief COPE		X	X	X
Family Nutrition and Physical Activity Screening Tool		X	X	X
Household Food Security Survey		X	X	X

Note:

* Primary outcome; PKBS-2, Preschool and Kindergarten Behavior Scales-Second Edition; BMI, body mass index; PHQ-4, Patient Health Questionnaire; MAAS, Mindful Attention Awareness Scale; CPRS, Child-Parent Relationship Scale.

## Data Availability

No data was used for the research described in the article.
